# Association between hospital volume and network membership and an analgesia, sedation and delirium order set quality score: a cohort study

**DOI:** 10.1186/cc11390

**Published:** 2012-06-18

**Authors:** Christopher R Dale, Shailaja J Hayden, Miriam M Treggiari, J Randall Curtis, Christopher W Seymour, N David Yanez, Vincent S Fan

**Affiliations:** 1 Division of Pulmonary and Critical Care Medicine, University of Washington, 329 9th Avenue, Seattle, WA 98104, USA; 2 Department of Anesthesiology and Pain Medicine, University of Washington, 329 9th Avenue, Seattle, WA 98104, USA; 3 Department of Critical Care, University of Pittsburgh School of Medicine, 3550 Terrace Street, Pittsburgh, PA 15261, USA; 4 Department of Biostatistics, University of Washington, 1705 NE Pacific Street, Seattle, WA 98195, USA; 5 Division of Pulmonary and Critical Care Medicine, University of Washington, 1100 Olive Way, Suite 1400, Seattle, WA 98108, USA

## Abstract

**Introduction:**

Protocols for the delivery of analgesia, sedation and delirium care of the critically ill, mechanically ventilated patient have been shown to improve outcomes but are not uniformly used. The extent to which elements of analgesia, sedation and delirium guidelines are incorporated into order sets at hospitals across a geographic area is not known. We hypothesized that both greater hospital volume and membership in a hospital network are associated with greater adherence of order sets to sedation guidelines.

**Methods:**

Sedation order sets from all nonfederal hospitals without pediatric designation in Washington State that provided ongoing care to mechanically ventilated patients were collected and their content systematically abstracted. Hospital data were collected from Washington State sources and interviews with ICU leadership in each hospital. An expert-validated score of order set quality was created based on the 2002 four-society guidelines. Clustered multivariable linear regression was used to assess the relationship between hospital characteristics and the order set quality score.

**Results:**

Fifty-one Washington State hospitals met the inclusion criteria and all provided order sets. Based on expert consensus, 21 elements were included in the analgesia, sedation and delirium order set quality score. Each element was equally weighted and contributed one point to the score. Hospital order set quality scores ranged from 0 to 19 (median = 8, interquartile range 6 to 14). In multivariable analysis, a greater number of acute care days (*P *= 0.01) and membership in a larger hospital network (*P *= 0.01) were independently associated with a greater quality score.

**Conclusions:**

Hospital volume and membership in a larger hospital network were independently associated with a higher quality score for ICU analgesia, sedation and delirium order sets. Further research is needed to determine whether greater order-set quality is associated with improved outcomes in the critically ill. The development of critical care networks might be one strategy to improve order set quality scores.

## Introduction

In the ICU setting, mechanical ventilation is common, resource intensive and associated with pain, anxiety and delirium [1,2]. Protocols and order sets (the mechanism by which a protocol is implemented) have been developed to optimize the care of mechanically ventilated patients [3]. In fact, analgesia and sedation protocols improve the duration of mechanical ventilation, the length of ICU stay, and survival in the setting of clinical trials [4-7]. However, the implementation of these protocols and their content are variable. Recent surveys showed that just over 70% of American academic ICUs had sedation protocols and only 40% of Canadian intensivists used a daily spontaneous awakening trial, an intervention shown to decrease the duration of mechanical ventilation [5,8-10].

Clinical practice guidelines for mechanically ventilated patients have been developed by international professional organizations based on the analgesia, sedation and delirium treatment literature [11,12]. Ideally, these guidelines and the professional literature itself would help hospitals develop order sets that would then be used in their ICUs to deliver evidence-based patient care. There is significant variation in order set development and guideline implementation, however, suggesting that this knowledge translation process is neither seamless nor uniform [9,13].

Order set creation and knowledge translation are complex and incompletely understood processes [14-16]. Provider work load, lack of resources, variability in interpretation, and lack of familiarity with the guidelines have all been cited as reasons for incomplete knowledge translation [3,13]. Using a structure-process-outcome model of quality improvement, we sought to identify hospital-level predictors of ICU analgesia, sedation and delirium order set quality - a structure quality element [17]. As an established relationship between volume and outcome exists in medicine, we hypothesized that higher-volume hospitals and hospitals that are members of a hospital network would be more likely to have higher-quality order sets with greater adherence to published guidelines [18-21]. We chose to look at hospital networks because some have described a potential role for the regionalization of critical care and our own experience has highlighted the fact that hospitals in a network tend to share protocols and order sets within their networks [22,23].

## Materials and methods

### Hospital sample

We conducted a cross-sectional study of all nonfederal, nonpediatric acute care hospitals in Washington State between December 2010 and June 2011. Hospitals were identified using the Washington State Department of Health hospital database as well as the Washington State Hospital Association hospital list. Representatives of ICU leadership in each hospital were interviewed to determine whether their hospital provided ongoing care (defined as care for > 24 hours) for mechanically ventilated patients. All interviews were completed by telephone by one investigator (CRD). We excluded hospitals that transferred patients requiring ongoing mechanical ventilation, even if they provided short-term care to postoperative ventilated patients and those patients requiring stabilization prior to transfer.

**Figure 1:**
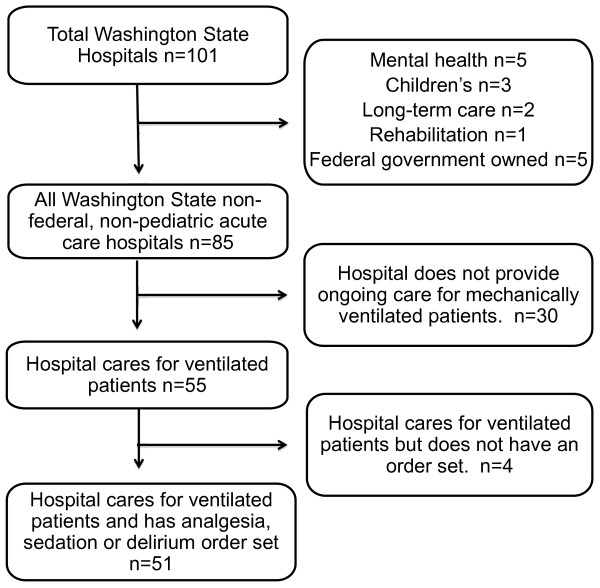
**Flowchart of the hospital cohort**.

A total of 101 hospitals were identified (Figure 1). Of these, 85 were nonfederal, nonpediatric acute care hospitals. Thirty hospitals did not provide ongoing care for ventilated patients and were excluded. Twenty-nine of the 30 acute care hospitals (97%) that did not provide ongoing mechanical ventilation were critical access hospitals, which by definition have 25 beds or fewer. Fifty-five hospitals provided ongoing care for ventilated patients. A member of the ICU leadership team at each of these hospitals was interviewed to determine whether the hospital possessed an order set or protocol that could be used to provide analgesia, sedation or delirium treatment for ICU patients. Fifty-one of the 55 hospitals (93%) reported that they had an order set or protocol. All provided a copy of their order sets and/or protocols and were included in the study sample. The four hospitals that provided ongoing care to mechanically ventilated patients but did not have an order set for analgesia, sedation or delirium treatment were excluded from the analysis. These four excluded hospitals had a median of 52 acute care beds and 21,132 acute care days in 2009, and one was a critical access hospital. Hospitals with multiple ICUs were queried about the existence of multiple order sets in their various ICUs, but all reported using the same analgesia, sedation and delirium protocol in all of their ICUs.

The Human Subjects Division of the University of Washington reviewed the study and determined it not to be human subject research and therefore exempt.

### Variable definitions and abstraction

Hospital characteristics - including acute care and ICU bed numbers, acute care and ICU days, critical access status and hospital governance structure - were obtained from the Washington State Department of Health database for 2009. The ICU leadership team at each hospital was interviewed to ascertain the number of ICUs at the hospital, the availability of computerized provider order entry (CPOE), whether resident physicians provided care in the ICU, and whether the ICU was open or closed. A closed ICU was defined as one where all patients had their care transferred to, or directed by, an intensivist-led team [24]. A hospital network was defined as an entity that operated two or more hospitals in Washington State or an adjoining metro area. The hospital's membership in a larger hospital network was determined by review of the 2008 American Hospital Association hospital data file and a structured web search, and was confirmed via the ICU leadership interview. The chosen reference date for membership in a hospital network and the presence of CPOE was 1 January 2011.

The ICU leadership team was asked for an electronic or faxed copy of the order set or protocol that they used to provide analgesia, sedation and/or delirium management for mechanically ventilated patients. If more than one protocol was used to provide analgesia, sedation and delirium care (for example, a separate delirium protocol) then all relevant protocols were obtained and analyzed as one.

### Development of an analgesia, sedation and delirium quality score

The 2002 American College of Critical Care Medicine, American Society of Health-System Pharmacists and American College of Chest Physician clinical practice guidelines for the sustained use of sedatives and analgesics in the critically ill adult were reviewed to generate a list of possible components that a high-quality analgesia, sedation and delirium protocol could contain (Table 1) [11]. Twenty-four proposed quality elements were found to be unique indicators of order set quality (10 items pertaining to analgesia, 10 items to sedation, and four items pertaining to delirium). Two potentially significant elements were not addressed by the 2002 guidelines: a daily spontaneous awakening trial for each eligible patient, and a paired daily spontaneous awakening trial and spontaneous breathing trial for all eligible patients. We felt that these elements were important enough to merit consideration for inclusion in the quality score even though they were not included in the 2002 guidelines. We purposely did not included elements related to specific medications or assessment tools.

**Table 1:** Proposed order set quality elements based on the 2002 sedation guidelines expert-validated quality score

Analgesia elements (*n *= 10)	Expert quality score	Sedation elements (*n *= 10)	Expert quality score	Delirium elements (*n *= 4)	Expert quality score
Pain is regularly assessed	1.0 (0)	Sedation is regularly assessed	1.0 (0)	Delirium is regularly assessed	1.7 (0.72)
Pain is regularly documented	1.0 (0)	Sedation is regularly documented	1.0 (0)	Delirium is treated on a symptom-oriented basis	1.9 (0.64)
Validated pain scale is used to assess pain	1.2 (0.56)	Validated sedation assessment scale is used	1.1 (0.26)	Antipsychotic medications are included in the order set for the pharmacologic treatment of delirium	1.7 (0.72)
Self-report of pain is used to assess pain	1.2 (0.56)	Sedation goal or endpoint is established for each patient	1.0 (0)	*The QT interval is monitored in patients receiving antipsychotics*^a^	1.8 (0.77)
Therapeutic goal of analgesia is established	1.1 (0.35)	The sedative dose is titrated to a defined endpoint	1.0 (0)		
The analgesia goal is patient specific	1.0 (0)	Sedation of agitated critically ill patients is started only after providing adequate analgesia	1.4 (0.63)		
Analgesic medications are titrated based on the patient's goal and plan	1.0 (0)	Sedation of agitated critically ill patients is started only after treating reversible physiologic causes	1.4 (0.63)		
NSAIDs or acetaminophen should be provided as optional adjuncts to opioids in selected patients	2.0 (0.76)	A sedation algorithm or protocol is used to guide the administration of sedation	1.2 (0.41)		
*Scheduled opioid doses or a continuous infusion are given preferentially over an as-needed regimen*	2.7 (1.1)	Daily interruption of sedation is performed in all patients who lack a contraindication	1.1 (0.26)		
*A patient-controlled analgesia device is preferred to deliver opioids if the patient is able to understand and operate the device*	2.1 (0.74)	The daily sedation interruption is explicitly linked to a spontaneous breathing trial	1.4 (0.51)		

Data presented as mean (standard deviation). NSAID, nonsteroidal anti-inflammatory drug. The expert quality score ranged from 1 (all high-quality analgesia/sedation order sets should contain this element) to 4 (a high-quality analgesia/sedation order set would not contain this element). Elements with an average rating of 2 or better were included in the order set quality score. Elements in italics were not included in the final order set quality score. ^a^Hospitals without antipsychotics on their order sets may not require this element.

A web-based survey was then sent to a convenience sample of 20 North American ICU directors and experts - individuals who have been academically active in ICU analgesia, sedation and delirium work - asking them to rate the importance of the 24 potential quality elements on the following four-point scale: 1 = very important (all high-quality critical care analgesia, sedation and delirium order sets should contain this element); 2 = somewhat important (a high-quality critical care analgesia/sedation order set would most probably contain this element, but some high-quality order sets may not contain it); 3 = not important/optional (a high-quality critical care analgesia/sedation order set may or may not contain this element); and 4 = drop/do not include (a high-quality critical care analgesia, sedation and delirium order set would not contain this element).

Of the 20 experts in ICU analgesia, sedation and delirium, 15 (75%) completed the survey and their responses were used to create a quality score. The mean (standard deviation) scores are shown in Table 1. All elements with a mean rating that the element would more likely than not be included in a high-quality order set were included in our score. Two items that were rated so as not to be included were: scheduled opioid doses or a continuous infusion are given preferentially over an as-needed regimen; and a patient-controlled analgesia device is preferred to deliver opioids if the patient is able to understand and operate the device.

To score the order sets and compute the quality score, composite survey questions with an 'and' or an 'or' conjunction were separated into two elements for abstraction. For example, 'pain is regularly assessed and documented' was abstracted as two separate items. The element 'the QT interval is monitored in patients receiving antipsychotics' was excluded from the final quality score as not all hospitals had antipsychotics as part of their orders and therefore may not require this quality element. Each element, regardless of its expert rating, was assigned one point towards the total score. The highest possible quality score was 21.

After training on a collection of order sets from non-Washington State hospitals, the contents of the 51 hospital order sets were all independently abstracted by two researchers (CRD and SJH) There was initial disagreement on only 189 of the 1,224 total possible elements (85% agreement). We were able to come to consensus regarding 100% of the elements after discussion of each initial difference.

### Analysis

The hospital characteristics were summarized using percentages or medians (interquartile range), and were compared between quartiles of order set quality score. Domains of the quality score were summarized using medians (interquartile range), and were compared along quartiles of number of acute care hospital days. The number of ICU beds, the number of ICU bed-days, the total hospital beds and the total hospital days were all highly correlated. The number of acute care bed-days was chosen to represent hospital volume, and the number of ICU bed-days was chosen to represent ICU volume [18,19].

We used multivariable clustered linear regression to model the association between hospital-level factors and the order set quality score. We chose hospital-level factors *a priori *based on the literature and included the following: teaching hospital status, CPOE status, membership in a hospital network, number of acute care bed-days, number of ICU bed-days, hospital organizational structure, critical access status, and open versus closed ICU model. Our final model clustered on order sets with common elements within a hospital network. We observed collinearity between acute care bed-days and ICU bed-days as assessed by the variance inflation factor, and ultimately excluded ICU bed-days from the final model. Finally, we performed a number of sensitivity analyses, examining alternative definitions of hospital volume and the order set quality score to test the robustness of our findings. *P *< 0.05 was selected to denote statistical significance. All statistical tests were two-sided and performed using STATA version 11.1 (StataCorp, College Station, TX, USA).

## Results

The results of the expert survey are summarized in Table 1. There were several elements about which there was consensus that they were very important elements of a high-quality order set, including assessing and documenting both pain and sedation and titrating analgesia and sedation medications based on a patient's goal (pain) or a defined endpoint (sedation). The experts rated the analgesia and delirium elements higher than the sedation ones, as indicated by a lower mean expert survey score for the sedation elements than the analgesia or delirium elements (*P *< 0.01 for both).

The characteristics of the 51 eligible hospitals are summarized in Table 2. Hospital bed-days per year ranged from 3,654 to 131,881 (median 31,011). We observed that higher volume hospitals were more likely to be members of a larger hospital network (*P *= 0.01) and more likely to be a teaching ICU (*P *< 0.01). Larger ICUs were less likely to be open (*P *= 0.01) and less likely to be a part of a critical access hospital (*P *= 0.02).

**Table 2:** Hospital characteristic by quartile of analgesia, sedation and delirium order set quality score

Hospital characteristic	All hospitals (*n *= 51)	Score 0 to 6 (*n *= 17)	Score 7 to 8 (*n *= 9)	Score 9 to 14 (*n *= 16)	Score 15 to 19 (*n *= 9)
Number of hospital beds	107 (44 to 198)	33 (21 to 69)	143 (101 to 198)	122 (96 to 122)	250 (156 to 315)
Number of hospital days 2009	31,011 (6,974 to 59,696)	5,979 (4,061 to 14,265)	33,964 (19,612 to 54,391)	34,828 (24,480 to 48,239)	79,759 (34,446 to 108,542)
Number of ICU beds	16 (6 to 31)	6 (4 to 9)	16 (15 to 20)	20 (10 to 31)	38 (27 to 80)
Number of ICU days 2009	3,772 (1,284 to 8,603)	1,200 (630 to 3,772)	3,759 (3,224 to 4,784)	5,678 (2,588 to 9,040)	18,623 (8,219 to 31,715)
Part of a larger hospital network	25 (49.0)	4 (23.5)	2 (22.2)	10 (62.5)	9 (100)
Critical access hospital	8 (15.7)	7 (41.2)	0 (0)	1 (6.3)	0 (0)
Private, not-for-profit hospital	30 (58.8)	7 (41.2)	4 (44.4)	13 (81.2)	6 (66.7)
Public, not-for-profit hospital	17 (33.3)	10 (58.8)	2 (22.2)	3 (18.8)	2 (22.2)
Private, for-profit hospital	4 (7.8)	0 (0)	3 (33.3)	0 (0)	1 (11.1)
Number of ICUs	1 (1 to 1)	1 (1 to 1)	1 (1 to 1)	1 (1 to 1)	2 (2 to 2)
Teaching ICU	16 (31.4)	2 (11.8)	3 (33.3)	4 (25.0)	7 (77.8)
Open ICU	46 (90.2)	17 (100)	8 (88.9)	15 (93.8)	6 (66.7)
Computerized provider order entry	12 (23.5)	2 (11.8)	2 (22.2)	2 (12.5)	6 (66.7)

Data presented as median (interquartile range) or *n *(%).

We present the distribution of the analgesia, sedation and delirium quality score by network status and quartile of hospital volume in Table 3. The median total quality score was 8 (interquartile range 6 to 14; range 0 to 19) and was approximately normally distributed. Higher hospital volume was associated with higher quality scores in bivariable analysis (*P *< 0.01). We also observed that the association between quartile of number of ICU beds and quality score was consistent across each of the separate analgesia, sedation and delirium domains (*P *< 0.01 for all; Table 3).

**Table 3:** Analgesia, sedation and delirium order set quality score by network status and acute care days

	Network status				
		
Quality score domain	All hospitals (*n *= 51)	Not part of a network (*n *= 26)	Part of a network (*n *= 25)	*P *value^a^	
Analgesia	2 (0 to 4)	0 (0 to 2)	4 (1 to 5)	< 0.01	
Sedation	6 (5 to 8)	6 (4 to 6)	8 (6 to 9)	< 0.01	
Delirium	0 (0 to 2)	0 (0 to 1)	2 (0 to 3)	< 0.01	
Total quality score	8 (6 to 14)	6 (5 to 8)	13 (9 to 15)	< 0.01	
	
	**Quartile of acute care days (2009)**				
	
	**3,654 to 6,974 days (*n *= 13)**	**8,614 to 14,265 days (*n *= 13)**	**31,579 to 59,696 days (*n *= 13)**	**60,620 to 131,881 days (*n *= 12)**	***P *value for trend^b^**
	
Analgesia	0 (0 to 0)	2 (0 to 4)	2 (2 to 4)	4 (1 to 6)	< 0.01
Sedation	5 (2 to 6)	6 (5 to 7)	6 (5 to 7)	8 (6 to 9)	< 0.01
Delirium	0 (0 to 0)	0 (0 to 2)	2 (0 to 2)	2 (1 to 3)	< 0.01
Total quality score	5 (2 to 6)	8 (6 to 13)	9 (8 to 13)	14 (9 to 17)	< 0.01

Data are presented as median (interquartile range). ^a^*P *values obtained using a *t *test with unequal variance. ^b^*P *values obtained using clustered bivariable linear regression.

**Figure 2:**
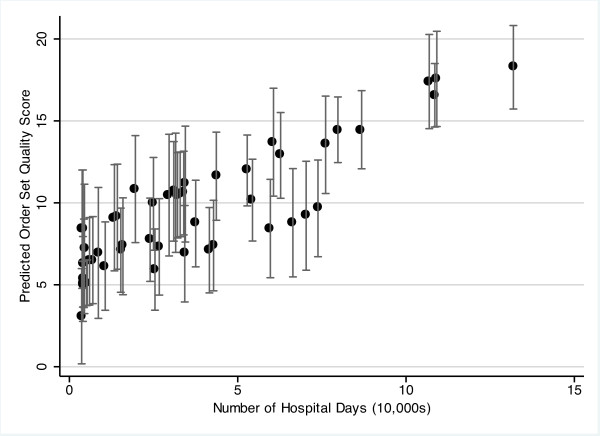
**Predicted order set quality score across number of hospital days, adjusted for measured hospital characteristics**. Predicted order set quality score across the number of hospital days for all hospitals (*n *= 51), adjusted for all measured hospital characteristics. Error bars correspond to 95% confidence intervals. The maximum possible score was 21.

The results of the multivariable model are shown in Table 4. After adjusting for all of the *a priori *identified potential confounders - including critical access status, open ICU status, teaching ICU status, CPOE and organizational structure - an increased hospital volume and membership in a hospital network were both associated with a higher quality score. A 100,000 patient-day increase in hospital volume was associated with an adjusted average quality score more than seven points higher (*P *< 0.01). The predicted quality scores were plotted to show the association between hospital volume and adjusted quality score (Figure 2).

**Table 4:** Association between hospital factors and order set quality score in clustered multivariable linear regression model

Hospital factor	Regression coefficient, β (95% confidence interval)	*P *value
10,000 hospital patient-days	0.73 (0.2, 1.2)	< 0.01
Part of a larger network	4.04 (1.2, 6.9)	< 0.01
Critical access hospital	-1.25 (-4.1, 1.6)	0.38
Teaching ICU	0.19 (-2.6, 3.0)	0.89
Open ICU	0.65 (-2.0, 3.3)	0.63
Computerized provider order entry	0.32 (-2.3, 3.0)	0.81
Private, not-for-profit hospital^a^	-1.9 (-4.8, 0.9)	0.18
Private, for-profit hospital^a^	-0.63 (-4.2, 2.9)	0.72

Model clustered on a similar order set within a network. ^a^Relative to public hospital status.

We also observed that membership in a hospital network was independently associated with a higher order set quality score. Hospitals that were members of a hospital network had order set quality scores that were more than four points higher, on average, than hospitals who did not participate in a network (*P *< 0.01).

We performed several sensitivity analyses. First, to address the concern that a quality score of equally weighted elements might simply reward length, we recalculated the expert quality score, assigning a greater weight to those elements with a lower (better) mean expert score. We assigned two points to those elements with a mean score of 1.2 to 1.5, and three points to those elements with a mean score < 1.2. Assigning this greater weight did not change our results. Second, we looked at different ways to model hospital volume, including ICU bed-days, number of ICU beds and number of hospital beds and their log-transformation - the results were similar, although not all reached statistical significance in the model. Finally, we included the four hospitals without an order set, assigning them a quality score of zero, and our results were unchanged.

From a qualitative perspective, order sets varied considerably. For example, one extreme featured only limited information on propofol administration. The other extreme featured complex algorithms and if-then algorithmic statements with comprehensive pain, agitation and delirium assessment and treatment.

## Discussion

We found among all Washington State hospitals that provide care for mechanically ventilated patients that greater hospital volume and membership in a hospital network were independently associated with higher quality scores for ICU analgesia, sedation and delirium order sets. To our knowledge this is the first rigorous assessment of the content of ICU analgesia, sedation and delirium order sets and the association between hospital-level factors and order set quality.

The development of order sets and their subsequent implementation is complex and poorly understood [3]. Landmark or significant clinical trials often form the foundation of clinical practice guidelines [25-28]. Ideally, hospitals incorporate the content of these guidelines or the results of the trials themselves into their routine patient care activities as a structure element of quality improvement. Yet this may not happen in a uniform manner at all hospitals. In fact, recent evidence suggests that the care provided by ICU practitioners may be less adherent to guidelines than the providers believe it to be and may lag significantly behind the publication of research findings and clinical guidelines [13,29].

The guidelines upon which we based our order set quality score were published in 2002 and they may be outdated to some degree. However, there was expert consensus for our study in 2011 that all of the elements included in our score were at least, on average, more likely than not to be included in a high-quality ICU analgesia, sedation and delirium order set. We also included two elements (a daily spontaneous awakening trial for each eligible patient, and a paired daily spontaneous awakening trial and spontaneous breathing trial for all eligible patients) that were not included in the original guidelines but that we thought experts would probably agree ought to be included in a high-quality order set. Although new guidelines are forthcoming, our study demonstrates associations between order set quality and hospital characteristics based on currently available guidelines.

The broad distribution of the order set quality score is notable and represents a potential opportunity for quality improvement. Although we were not able to examine whether the order set quality score was associated with patient outcomes, the implementation of sedation, sepsis, ventilator-associated pneumonia and weaning protocols have all been associated with improved patient outcomes and decreased costs [30-39]. As such, we believe our findings are an important first step in improving the structure of the care environment, a step that could lead to improvements in both processes and outcomes.

There are several possible reasons why higher hospital volume was associated with higher-quality order set scores. First, there is an extensive body of work showing an association between volume and outcome in medicine [18-21]. Volume may be a proxy for resources to devote to structure and process quality improvement endeavors. Additionally, prior studies have shown that high-performing hospitals have a cooperative culture that works to improve the process of care delivery throughout the hospital [15,40]. Cooperative culture and effective quality improvement efforts require the dedicated work of a multidisciplinary team that has the time and resources to engage in a resource-intensive enterprise [3,14,41]. High-volume hospitals may be more able to devote nursing, physician and pharmacy resources to foster a cooperative culture focused on quality improvement [42].

Membership in a hospital network was also associated with an increase in order set quality score. We hypothesize that hospital networks may be more likely to share protocols and order sets as well as be more likely to have the overall resources to devote to quality improvement activities, such as order set creation. Indeed, several of the hospital networks did have identical or nearly identical protocols in at least some of their hospitals. Hospital networks may also have more resources to devote to quality improvement across their broad population of hospitals, including the development and implementation of high-quality order sets. However, as the recent trial by Scales and colleagues showed, a network of hospitals for quality improvement purposes need not be constrained by common ownership, as we defined it in this study [41]. Furthermore, geographic networks, like the Keystone Project in Michigan, or participation in networks defined by common interest, like the Institute for Healthcare Improvement, have improved ICU outcomes [43,44]. Taken together, sharing resources such as protocols and order sets across networks may represent an opportunity for maximizing quality improvement. The development of critical care networks might present an opportunity to improve structure quality elements [22,23].

Interestingly, we did not observe an association between certain hospital characteristics and order set quality. Specifically, a hospital's ICU staffing model (open vs. closed) or the teaching status of the ICU were not independently associated with its order set quality score. This could partly be a function of our sample size of 51 hospitals or of the collinearity among these variables, as both had a bivariable association with the order set quality score.

Our study has some important limitations. First, we did not have access to patient-level or hospital-level process or outcome data and were unable to study the association between order set quality and clinical process or patient outcomes. Second, we used an expert-validated measure of order set quality, which will require external validation in future studies. Third, we weighted all elements in our quality score equally because our quality score was not designed to distinguish between the individual elements. However, specific elements (for example, a daily interruption of sedation) may be more strongly contributing to improved patient outcomes than others, and may be dependent upon other factors such as nurse-patient ratios or pharmacist staffing that we were not able to study [5,11]. Future studies with larger sample sizes will be required to understand how alternative weighting of quality score elements affects score performance. Fourth, our study was not designed to assess the process of order set creation, a topic that merits further study. Finally, our results derive from a single state involving hospital data from 2009 and may not be generalizable to other regions. However, we did study the 13th most populated US state and included all hospitals that cared for mechanically ventilated patients.

## Conclusions

Among hospitals that provide ongoing care to mechanically ventilated patients, a greater hospital volume and membership in a hospital network were both independently associated with a greater expert-validated order set quality score. Future research should focus on the relationship between order set quality scores and process and outcome measures of quality. Equally importantly, steps should be taken to improve the process of order set development and dissemination to help all hospitals obtain high-quality order sets. The development of regional critical care networks might be one way in which structure elements of quality could be improved.

## Key messages

• An expert-validated quality score was created to summarize the guideline concordance of all analgesia, sedation and delirium order sets in Washington State.

• Order sets are a structure element of a structure-process-outcome quality improvement model.

• Hospital volume and membership in a hospital network are both associated with a better analgesia, sedation and delirium order set quality score.

• The creation of networks of hospitals might be one way to improve a structure element of a structure-process-outcome model of ICU quality improvement.

• More research is needed to better understand the process of order set creation and dissemination and to better understand the relationship between order set quality scores and outcome measures of quality.

## Abbreviations

CPOE: computerized physician order entry.

## Competing interests

The authors declare that they have no competing interests.

## Authors' contributions

CRD served as the primary author, conceived and designed the study protocol, collected and analyzed all of the data, wrote the manuscript and its revisions, and approved the final version of the manuscript. SJH designed the study protocol, collected and analyzed the data, reviewed the manuscript and approved the final version of the manuscript. MMT designed the study protocol, analyzed study data, reviewed the manuscript and approved the final version of the manuscript. JRC designed the study protocol, collected and analyzed study data, reviewed the manuscript and approved the final version of the manuscript. CWS collected and analyzed the study data, reviewed the manuscript and approved the final version of the manuscript. NDY III analyzed the study data, reviewed the manuscript and approved the final version of the manuscript. VSF designed the study protocol, analyzed the data, reviewed the manuscript and approved the final version of the manuscript.
